# Gene co-expression network analysis reveals key pathways and hub genes in Chinese cabbage (*Brassica rapa* L.) during vernalization

**DOI:** 10.1186/s12864-021-07510-8

**Published:** 2021-04-06

**Authors:** Yun Dai, Xiao Sun, Chenggang Wang, Fei Li, Shifan Zhang, Hui Zhang, Guoliang Li, Lingyun Yuan, Guohu Chen, Rifei Sun, Shujiang Zhang

**Affiliations:** 1 Institute of Vegetables and Flowers, Chinese Academy of Agricultural Sciences, Beijing, 100081 China; 2 College of Horticulture, Vegetable Genetics and Breeding Laboratory, Anhui Agricultural University, Changjiang West Road, NO.130, Hefei, 230036 Anhui China

**Keywords:** Chinese cabbage, Gradient-vernalization, RNA sequencing, Weighted gene co-expression network analysis, Hub genes

## Abstract

**Background:**

Vernalization is a type of low temperature stress used to promote rapid bolting and flowering in plants. Although rapid bolting and flowering promote the reproduction of Chinese cabbages (*Brassica rapa* L. *ssp. pekinensis*), this process causes their commercial value to decline. Clarifying the mechanisms of vernalization is essential for its further application. We performed RNA sequencing of gradient-vernalization in order to explore the reasons for the different bolting process of two Chinese cabbage accessions during vernalization.

**Results:**

There was considerable variation in gene expression between different-bolting Chinese cabbage accessions during vernalization. Comparative transcriptome analysis and weighted gene co-expression network analysis (WGCNA) were performed for different-bolting Chinese cabbage during different vernalization periods. The biological function analysis and hub gene annotation of highly relevant modules revealed that shoot system morphogenesis and polysaccharide and sugar metabolism caused early-bolting ‘XBJ’ to bolt and flower faster; chitin, ABA and ethylene-activated signaling pathways were enriched in late-bolting ‘JWW’; and leaf senescence and carbohydrate metabolism enrichment were found in the two Chinese cabbage-related modules, indicating that these pathways may be related to bolting and flowering. The high connectivity of hub genes regulated vernalization, including *MTHFR2*, *CPRD49*, *AAP8*, endoglucanase 10, *BXLs*, *GATLs*, and *WRKYs*. Additionally, five genes related to flower development, *BBX32* (binds to the *FT* promoter), *SUS1* (increases *FT* expression), *TSF* (the closest homologue of *FT*), *PAO* and *NAC029* (plays a role in leaf senescence), were expressed in the two Chinese cabbage accessions.

**Conclusion:**

The present work provides a comprehensive overview of vernalization-related gene networks in two different-bolting Chinese cabbages during vernalization. In addition, the candidate pathways and hub genes related to vernalization identified here will serve as a reference for breeders in the regulation of Chinese cabbage production.

**Supplementary Information:**

The online version contains supplementary material available at 10.1186/s12864-021-07510-8.

## Background

Chinese cabbage (*Brassica rapa* L. *ssp. pekinensis*), also known as heading cabbage or wrapping cabbage, is a leafy *Brassica* vegetable of the cruciferous family that originated in China with a long history of cultivation. Chinese cabbage has the characteristics of a rich variety of types, wide distribution, high yield, durability during storage and transportation, and a long supply period, and it is both highly nutritious and deeply loved by consumers. Chinese cabbage is one of the most economically important *Brassica* vegetable crops cultivated in Asian countries [1]. In Europe, especially Western Europe, the area of land under cultivation for Chinese cabbage has increased [2]. This indicates that the demand for Chinese cabbage throughout the year is slowly increasing. However, Chinese cabbage is susceptible to low temperatures (vernalization) and long daylight hours during the spring cultivation process, which causes it to bolt and flower quickly, thereby losing its commercial value. In contrast, in the breeding process, low temperature (vernalization) can be used to rapidly breed excellent varieties.

The transition from vegetative to reproductive growth is an important developmental step in the plant life cycle [3], and the timing of this switch is crucial for successful reproduction [4]. Vernalization, the effect of low temperature that induces and promotes flowering, is the main factor that promotes the transition from vegetative to reproductive growth in some biennial plants and annual winter plants. If plants that require low-temperature treatment do not undergo proper vernalization, flowering will be delayed by a few weeks or flower primordia will not form and will gradually decline. Different plants have different vernalization requirements depending on the developmental stage, vernalization temperature, and length of vernalization [5]. Previously, Yui and Yoshikawa [6] observed the phenomenon of low temperature promoting Chinese cabbage bolting and flowering. In the vernalization pathway, FLOWERING LOCUS C (*FLC*) is a key gene that controls flowering time. Many upstream genes ultimately determine bolting and flowering time by regulating the expression of *FLC*. *FLC* encodes a MADS-box transcription factor, which is a flowering inhibitor. The difference between early and late flowering depends largely on *FLC* allele variation [7]. FRIGIDA (*FRI*) is required for high *FLC* expression levels in Chinese cabbage and is a positive regulator of *FLC* [8]. Vernalization inhibits the expression of *FLC* and promotes flowering, and the dominant *FRI* allele strengthens the inhibition of *FLC* [9, 10]. The vernalization of Chinese cabbage also involves the expression of *VIN3*, *VRN2*, and *VRN1* [11]. Among them, *VRN1* and *VRN2* inhibit the expression of *FLC* and maintain the state of vernalization. Moreover, *VRN1* and *VRN2* do not recover after vernalization and maintain a continuous low expression state. *VIN3* participates in inhibiting the expression of *FLC* in early vernalization under low temperature conditions. In Chinese cabbage, Li Z et al. cloned the homologous gene *BrpFLC* of *FLC* of *Arabidopsis* and proved that different degrees of vernalization can reduce the transcription level of *BrpFLC* in different bolting-resistant cabbage varieties [12]. So far, four *FLC* homologous genes (*BrFLC1*, *BrFLC2*, *BrFLC3*, and *BrFLC5*) have been found and verified in Chinese cabbage [13, 14]. Recently, *BrFLC5* has been proven to be a weakly regulated gene for flowering regulation in Chinese cabbage [15]. After years of research, genes including *FLC*, *VIN3*, and the *VRN* family are currently the most thoroughly studied genes related to vernalization in Chinese cabbage.

The transcriptome is used to study gene transcription in plant cells and the regulation of transcription overall. The application of RNA sequencing technology (RNA-Seq) has been widely used in various biological fields to explore various aspects of the life sciences. RNA-Seq has been widely used to study the related genes of many plants, including the characteristics of *Arabidopsis* [16], rice [17] and cucumber [18]. In a study on the vernalization of *Brassica*-type vegetables, Sun et al. [19] conducted a transcriptome analysis on pak choi (*Brassica rapa subsp. chinensis*) samples at different developmental stages after vernalized and control treatments to investigate differentially expressed genes (DEGs), and they found that *Bra014527*, *Bra024097*, and *Bra035940* exhibited obvious changes after vernalization. The homologous genes of these three genes also participated in the vernalization response of *Arabidopsis*. Therefore, it was speculated that these genes also responded to vernalization in pak choi. Qi et al. [20] used an RNA-Seq technology to obtain information including the DEGs, functional annotations, and variable shear, of Chinese cabbage samples before and after vernalization. Four candidate genes related to flowering were screened. As an important flowering crop, it is necessary to explore the underlying molecular mechanisms of flowering induction in Chinese cabbage.

Currently, vernalization is widely applied in vegetable production, especially in leafy vegetables. Spring Chinese cabbage lose their commercial value after premature bolting as a result of low-temperature effects. The length of breeding time is also shortened due to rapid bolting and flowering caused by vernalization. Therefore, the effects of vernalization on Chinese cabbage are worth dissecting and exploring. In this study, the gradient vernalization of two different bolting Chinese cabbage accessions were used to analyze the transcriptome pattern of Chinese cabbage during vernalization. Using a weighted gene co-expression network analysis (WGCNA), specific gene co-expression networks formed in Chinese cabbage during vernalization were identified in order to find the reasons for the different bolting.

## Results

### RNA sequencing and gene co-expression network construction

**Fig. 1:**
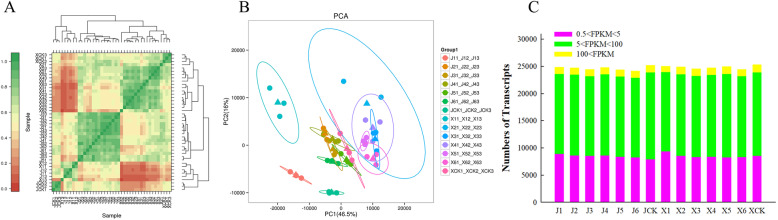
Transcriptional relationship between samples. **a** Heatmap of correlation value (R square) of 42 libraries. **b** Principal component analysis based on all of the expressed genes, showing 14 distinct groups of samples. **c** Number of transcripts in the 2 Chinese cabbage accessions, based on the FPKM of different samples

Pearson’s correlation coefficients were used to test for biologically repeated correlations between samples. The generated cluster dendrogram was used to observe the overall correlation of the transcriptomes of the 2 Chinese cabbage accessions at different time periods (Fig. 1a). The three biological replicates from each time period and the transcriptome data both exhibited good correlation. The similarity test between the three biological replicates required the use of a principal component analysis (PCA). Using the first principal component (PC1) and second principal component (PC2), a dimensionality reduction analysis was used to analyze the similarity between each replicate (Fig. 1b). A total of 14 groups exhibited good similarity. Approximately 59.37% of the expressed genes were within the 0–5 FPKM range and 37.36% were within the 5–100 FPKM range (Fig. 1c).

**Fig. 2:**
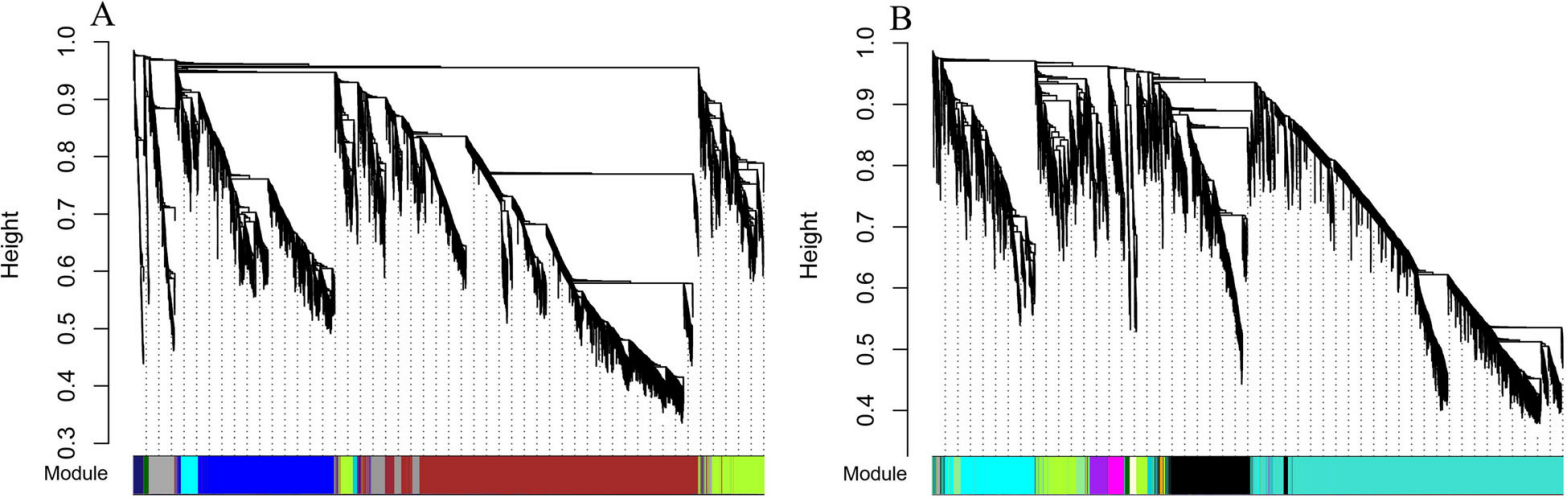
WGCNA of gene expression in ‘JWW’ **(a)** and ‘XBJ’ **(b)** during vernalization. Hierarchical cluster trees show the co-expression modules identified by WGCNA

**Fig. 3:**
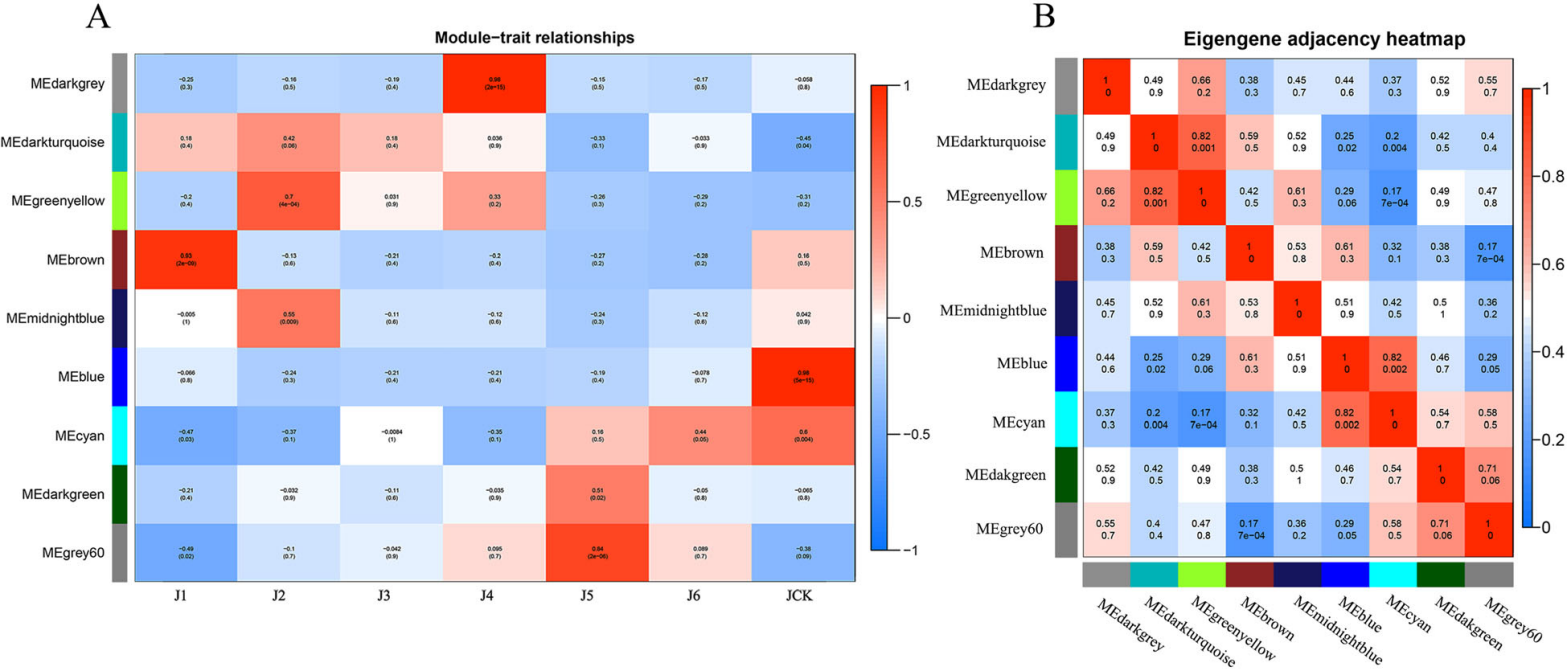
Co-expression modules for ‘JWW’. **a** Relationships between modules (left) and traits (bottom). Red and blue represent positive and negative correlations, respectively, with coefficient values and *p*-values. **b** Pairwise correlation coefficients between modules. Rows and columns are the module names, numbers represent coefficient values and *p*-values

**Fig. 4:**
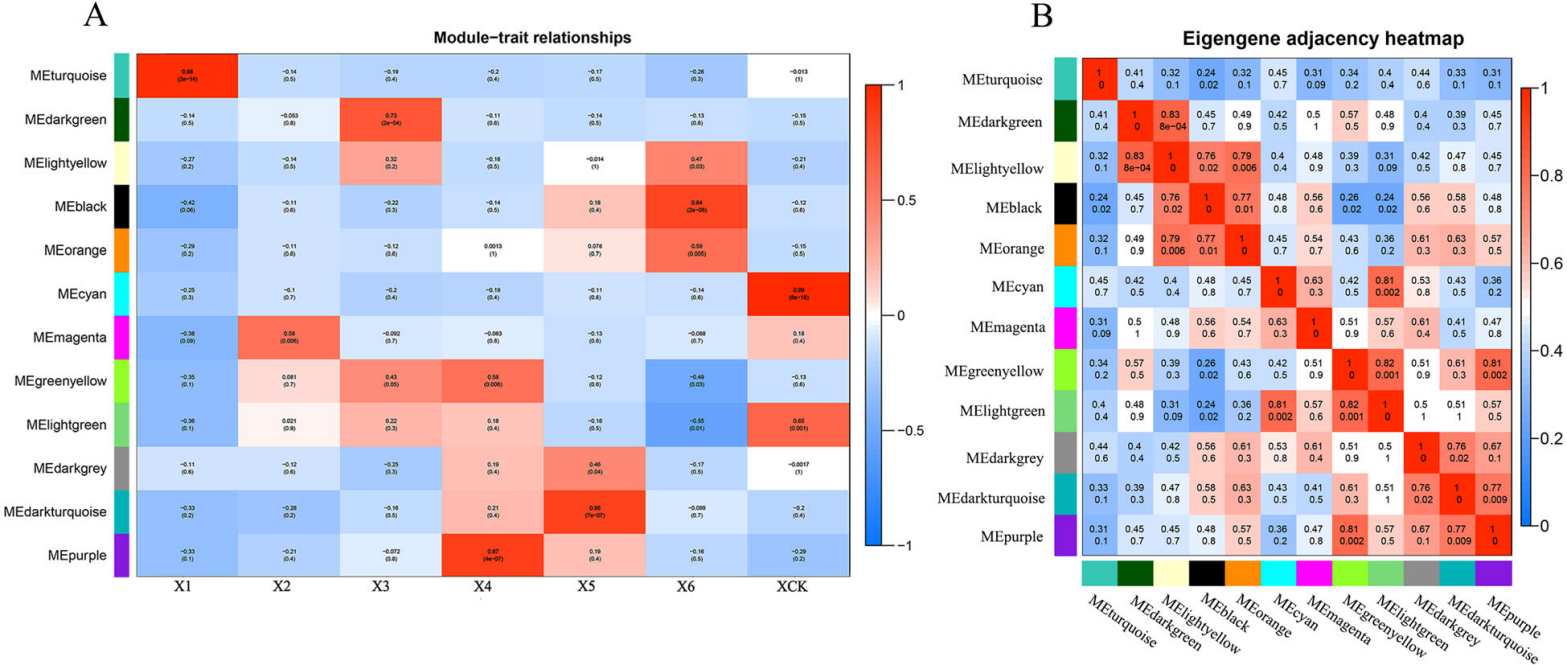
Co-expression modules for ‘XBJ’. **a** Relationships between modules (left) and Traits (bottom). Red and blue represent positive and negative correlations, respectively, with coefficient values and *p*-values. **b** Pairwise correlation coefficients between modules. Rows and columns are the module names, numbers represent coefficient values and *p*-values

After analyzing the transcriptome data of each treatment period of 2 Chinese cabbage accessions, low abundance and low variability genes were filtered out. A total of 5748 genes of ‘JWW’ and 5527 genes of ‘XBJ’ were screened out. After being log_2_-transformed, they were imported into the WGCNA software package for analysis. WGCNA analysis performed transcriptome data analysis in each period. Each tree branch formed a module and each leaf in the branch represented a gene, as shown in the hierarchical clustering tree (Fig. 2). Then, the tree from the dendrogram was cut into modules (clusters). Based on their correlation with vernalization and control time, sets of genes (modules) were identified. As shown in the tree dendrogram, WGCNA analysis resulted in 9 modules that were distinguishable by different colors for ‘JWW’; the number of target genes for each module ranged from 56 to 3685 (Table S1). WGCNA analysis resulted in 12 modules that were distinguishable by different colors for ‘XBJ’; the number of target genes for each module ranged from 36 to 3745 (Table S2). Each module corresponded to each period and had its correlation. Whether the correlation was positive or negative and the size of the correlation showed the degree of correlation with the target gene screened out by the transcriptome data of this period (Figs. 3 and 4a).

### Different modules related to ‘JWW’ and ‘XBJ’ in different periods

Module-trait relationships (MTRs) were different for each vernalization and control time period. These modules contained positively and negatively related genes, and their expression levels changed at different periods. Modules with MTR > 0.7 were selected as representatives of the 2 Chinese cabbage accessions for further analysis. Five modules were selected for both ‘JWW’ and ‘XBJ’. The results revealed the following high correlations: MEbrown (r = 0.93, *p* = 2e^− 09^) in J1 days after treatment (0 DAT); MEgreenyellow (r = 0.7, *p* = 4e^− 04^) in J2 (25 DAT); MEdarkgrey (r = 0.98, *p* = 2e^− 15^) in J4 (35 DAT); MEgrey60 (r = 0.84, *p* = 2e^− 06^) in J5 (45 DAT); MEblue (r = 0.98, *p* = 5e^− 15^) in JCK (35 DAT 25 °C) (Fig. 3a); MEturquoise (r = 0.98, *p* = 2e^− 14^) in X1 (0 DAT); MEdarkgreen (r = 0.73, *p* = 2e^− 04^) in X3 (15 DAT); MEpurple (r = 0.87, *p* = 4e^− 07^) in X4 (25 DAT); MEblack (r = 0.84, *p* = 2e^− 06^) in X6 (50 DAT); and MEcyan (r = 0.99, *p* = 8e^− 18^) in XCK (25 DAT 25 °C) (Fig. 4a).

**Fig. 5:**
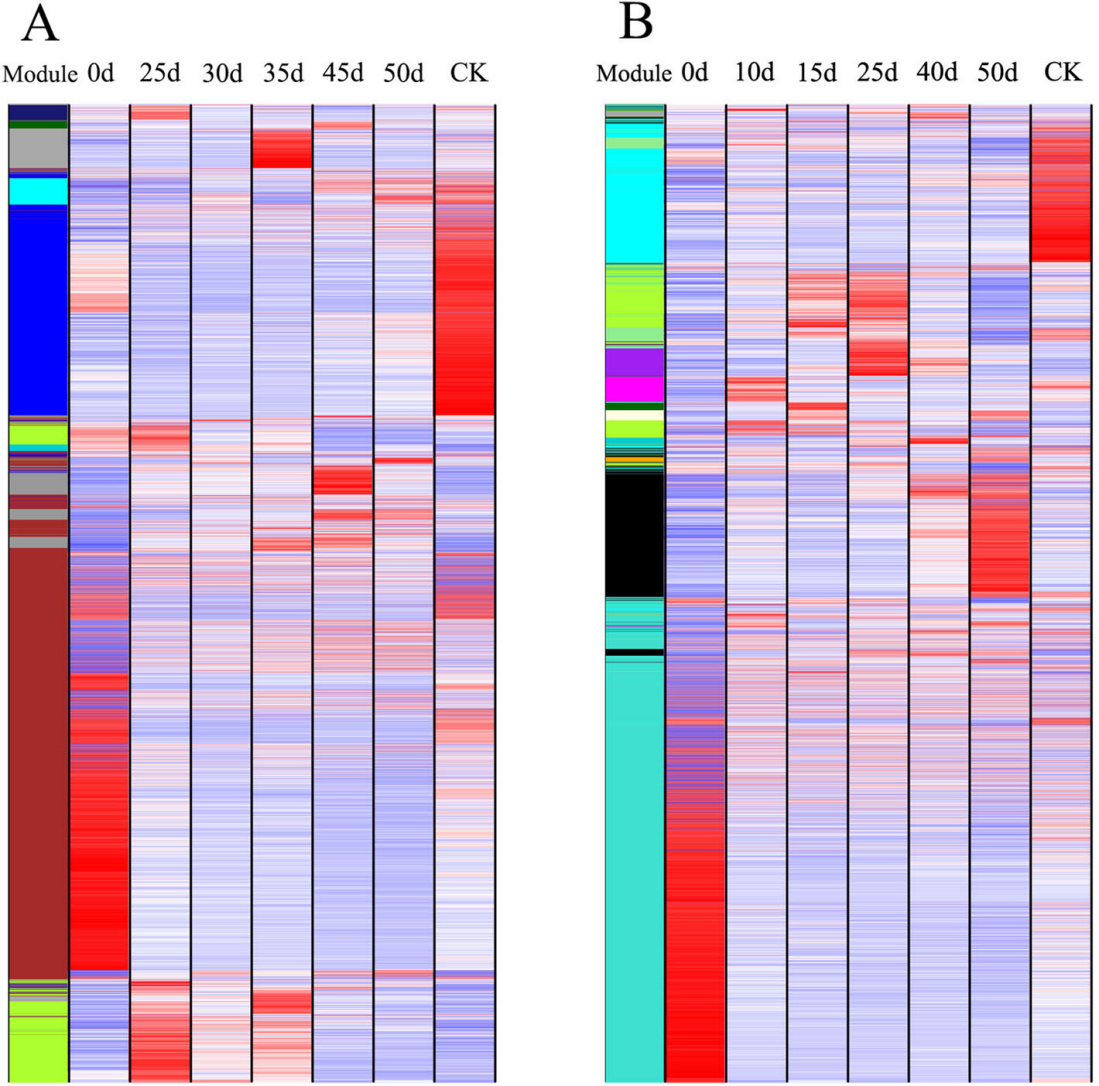
Gene expression levels in ‘JWW’ **(a)** and ‘XBJ’ **(b)** with their corresponding log_2_FPKM module values. The color gradient from blue to red indicates high to low gene expression

The correlations between different modules of the 2 Chinese cabbage accessions were further investigated. Based on the eigengenes of each module, some module pairs were found to be significantly positively correlated. In ‘JWW’, MEdarkturquoise was positively correlated with MEgreenyellow (r = 0.82, *p* = 0.001) and MEblue and MEcyan were positively correlated (r = 0.82, *p* = 0.002) (Fig. 3b). In ‘XBJ’, MElightyellow was positively correlated with MEdarkgreen (r = 0.83, *p* = 8e^− 04^), MEgreenyellow was positively correlated with MElightgreen (r = 0.82, *p* = 0.001) and MEpurple (r = 0.81, *p* = 0.002) and MElightgreen was positively correlated with MEcyan (r = 0.81, *p* = 0.002)), MElightgreen was positively correlated with MEcyan (r = 0.81, *p* = 0.002) (Fig. 4b). Expression gene displays were performed for each Chinese cabbage processing stage and corresponded with each module (Fig. 5). Results revealed that the enrichment and differential expression displays from the co-expression network modules exhibited similar characteristics.

### Biological function analysis of important co-expression network modules

**Fig. 6:**
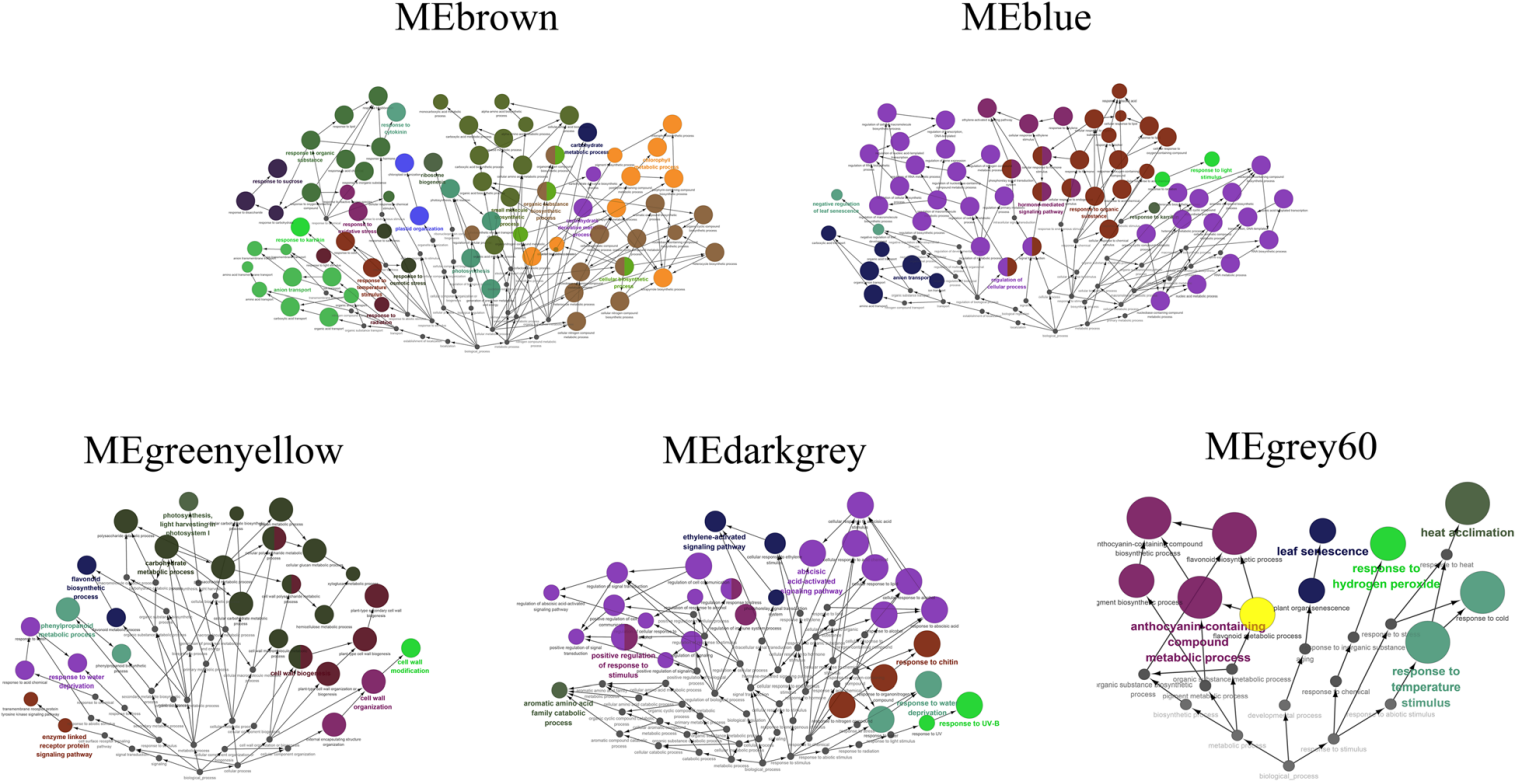
Significant GO terms and ontological relationships (annotated from ClueGO) in ‘JWW’. The sizes of the circles represent the degree of the positive relationship between the significant GO terms. Redundant terms were grouped and presented in the same color. Each leading term, which has the highest significance, is indicated by colored font

**Fig. 7:**
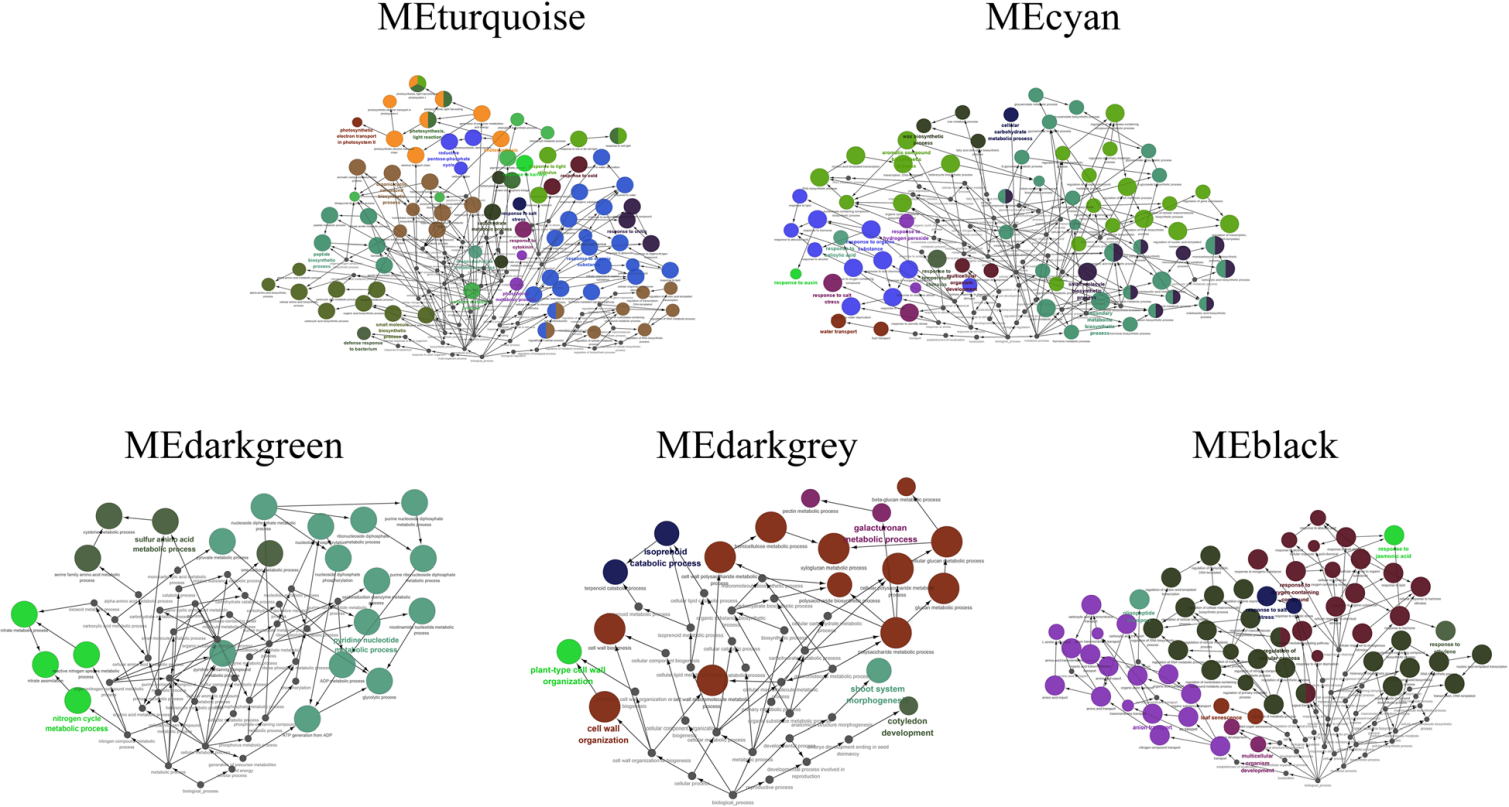
Significant GO terms and ontological relationships (annotated from ClueGO) in ‘XBJ’. The sizes of the circles represent the degree of the positive relationship between the significant GO terms. Redundant terms were grouped and presented in the same color. Each leading term, which has the highest significance, is indicated by colored font

GO annotations and biological function analysis were performed using 10 modules that were highly related (Figs. 6 and 7). *Brassica* genes were first used as queries. When the *Brassica* database was insufficient, *Arabidopsis* orthologue genes were used as queries. GO terms were derived from these annotations (Table S3; Table S4).

The biological functional terms enriched in ‘JWW’ MEbrown and ‘XBJ’ MEturquoise exhibited high correlation at 0 DAT and were the largest modules (*p* ≤ 0.01). In the *Brassica* database, ‘JWW’ MEbrown and ‘XBJ’ MEturquoise were enriched together with photosynthesis, response to cytokinin, chlorophyll biosynthetic process, and response to karrikin. The differences were ribosome biogenesis, translation, and response to unfolded protein, which were enriched in ‘JWW’ MEbrown, and light harvesting in photosystem I, protein-chromophore linkage, and reductive pentose-phosphate cycle, which were enriched in ‘XBJ’ MEturquoise. In the *Arabidopsis* Database, photosynthesis was the most enriched functional term in ‘JWW’ MEbrown and ‘XBJ’ MEturquoise. Additionally, cellular biosynthetic process, plastid organization, and anion transport were enriched in ‘JWW’ MEbrown, while cellular response to hormone stimulus, cellular response to endogenous stimulus, and cellular response to organic substance were enriched in ‘XBJ’ MEturquoise. These results indicated that the two Chinese cabbages had a certain degree of commonality to a large extent when they were not vernalized, and that when vernalized their different biological functions and gene expression might be observable.

‘JWW’ MEgreenyellow and ‘XBJ’ MEpurple were highly correlated at 25 DAT. The most enriched biological functional term in ‘JWW’ MEgreenyellow was cell wall organization in both the *Brassica* and *Arabidopsis* databases. In ‘XBJ’ MEpurple, the most enriched biological functional term in the *Brassica* database was xyloglucan metabolic process, while it was cell wall organization in the *Arabidopsis* database. In ‘JWW’ MEgreenyellow, several important biological functional terms were enriched, including cell wall biogenesis, carbohydrate metabolic process, and phenylpropanoid metabolic process. At 25 DAT, rapid flowering in ‘XBJ’ was promoted and was highly related to MEpurple. Biological functional terms related to polysaccharide metabolism processes were enriched, including polysaccharide metabolic process, cellular polysaccharide metabolic process, cell wall polysaccharide metabolic process, glucan metabolic process, cellular glucan metabolic process, and xyloglucan metabolic process. Additionally, shoot system morphogenesis was also enriched in this module. Thus, it was speculated that polysaccharide metabolism processes were enriched at 25 DAT in ‘XBJ’ to ensure that it transitioned from vegetative to reproductive growth, which was manifested by changes in shoot system morphogenesis.

‘JWW’ MEdarkgrey, which was highly correlated at 35 DAT, promoted rapid flowering and had many functional terms that were enriched in both databases, including response to water deprivation, response to chitin, abscisic acid (ABA)-activated signaling pathway, and response to UV-B. Additionally, response to stimulus, ethylene-activated signaling pathway, and aromatic amino acid family catabolic process, along with other terms, were positively regulated and enriched. These terms were enriched at 35 DAT during the critical vernalization period and may be the key biological functions that explain the transformation of late-bolting Chinese cabbage flowering.

MEdarkgreen, which was highly correlated with ‘XBJ’ at 15 DAT, was enriched in the functional terms nitric oxide biosynthetic process, glycolytic process, pyridine-containing compound metabolic process, sulfur amino acid metabolic process, and nitrogen cycle metabolic process, among other functional terms. The most enriched functional terms in ‘JWW’ MEgrey60 at 45 DAT included response to cold, circadian rhythm, response to temperature stimulus, and anthocyanin-containing compound metabolic process.

At 50 DAT, which was the largest vernalization period, ‘XBJ’ MEblack was enriched in functional terms related to hormones and amino acids, including response to ethylene, negative regulation of ethylene-activated signaling pathway, response to hormone, hormone-mediated signaling pathway, cellular response to hormone stimulus, amino acid export, and amino acid transmembrane transport. Additionally, reproductive growth and terms related to senescence were also enriched in this module, including positive regulation of leaf senescence, stress-induced premature senescence, and plant organ senescence.

‘JWW’ MEblue at 35 DAT at 25 °C, which was correlated with ‘JWW’ at 35 DAT in the control treatment, was enriched in the regulation of protein serine/threonine phosphatase activity, response to organic substance, hormone-mediated signaling pathway, and regulation of cellular process, among other functional terms. Notably, leaf senescence was negatively regulated and enriched in this module. Additionally, leaf senescence was positively regulated in ‘XBJ’ MEblack at 50 DAT, indicating that the leaf senescence of Chinese cabbage after vernalization may also signal bolting and flowering promotion. At 25 DAT, faster flowering was promoted in ‘XBJ’ MEcyan compared to 25 DAT at 25 °C, and ‘XBJ’ MEcyan was enriched in functional terms related to biosynthesis, including inositol biosynthetic process, aromatic compound biosynthetic process, small-molecule biosynthetic process, and wax biosynthetic process.

### Hub gene selection for the ‘JWW’ and ‘XBJ’ co-expression networks

**Fig. 8:**
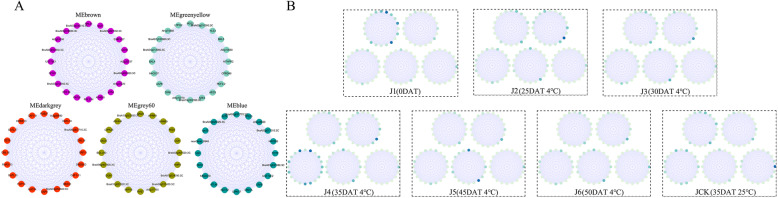
Hub genes and expression profiles of ‘JWW’. **a** Co-expression gene networks with the greatest “hubness” in every module. Nodes are represented by dots surrounded by module colors. **b** log_2_FPKM expression profiles of the hub genes in J1, J2, J3, J4, J5, J6, and JCK. The locations of each gene correspond with A. The darker the green color, the higher the expression level

**Fig. 9:**
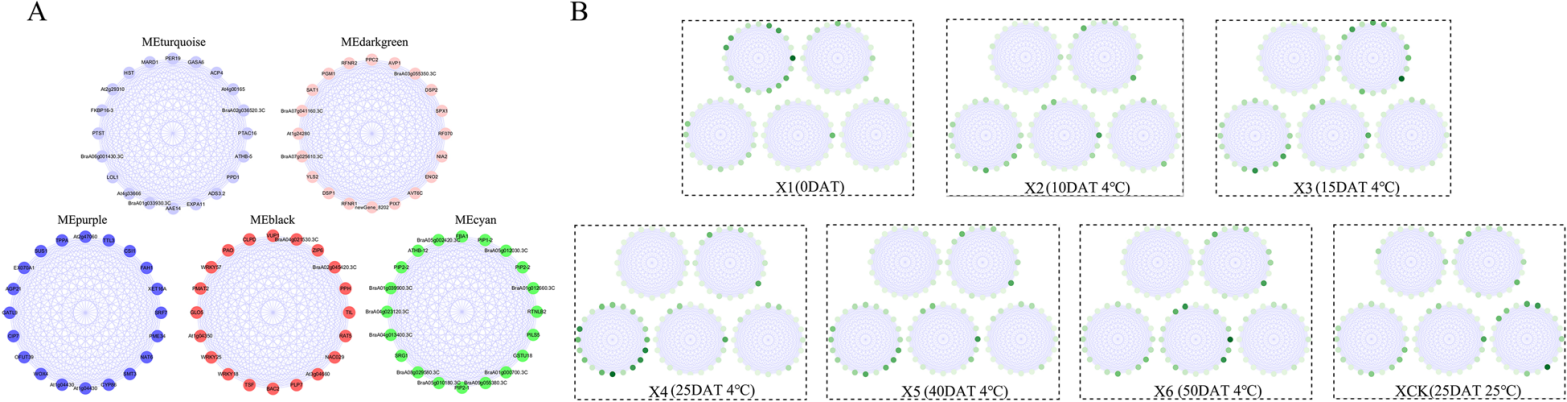
Hub genes and expression profiles of ‘XBJ’. **a** Co-expression gene networks with the greatest “hubness” in every module. Nodes are represented by dots surrounded by module colors. **b** log_2_FPKM expression profiles of the hub genes in X1, X2, X3, X4, X5, X6, and XCK. The locations of each gene correspond with A. The darker the green color, the higher the expression level

Hub genes were screened among these highly related modules across each time period. The top 20 genes that were representative of the modules were selected as they exhibited the largest “hubness” thereby providing the most detailed biological information (Figs. 8 and 9; Table S5; Table S6).

MEgreenyellow, MEdarkgrey, and MEgrey60 were highly related modules in ‘JWW’ across vernalization periods. For MEgreenyellow, methylenetetrahydrofolate reductase 2 (*MTHFR2*), GDSL esterase/lipase CPRD49 (*CPRD49*), and amino acid permease 8 (*AAP8*) were enriched in amino acid transport and metabolism pathways. Carbohydrate transport and metabolism pathways were enriched in 3 genes among the 20 hub genes, including endoglucanase 10, beta-D-xylosidase 4 (*BXL4*), and beta-D-xylosidase 5 (*BXL5*). Moreover, the expression levels of *MTHFR2*, *AAP8*, *BXL4*, and *BXL5* during vernalization were considerably higher than in the control treatment. Among the 20 genes expressed in MEdarkgrey: 3 glycosyl transferase family genes, *GATL10s*, and *GATL17* were the hub genes of MEdarkgrey and their expression levels were the highest in ‘JWW’ J4 (35 DAT), and may be important family genes that promote faster flowering in ‘JWW’. Four AP2 domain genes, dehydration-responsive element-binding protein 1C (*DREB1C*), ethylene-responsive transcription factor 11 (*RRF11*), dehydration-responsive element-binding protein 1D (*DREB1D*), and ethylene-responsive transcription *RAP2–13* were also hub genes found in this module. Notably, in MEdarkgrey, the B-box zinc finger protein 32 (*BBX32*) gene was enriched in biological functional terms related to flower development regulation. The top 20 hub genes in MEgrey60 included two 2-component response regulator-like *APRR9* genes that were enriched in circadian rhythm-plant pathways.

Modules highly related to the ‘XBJ’ vernalization periods included MEdarkgreen, MEpurple, and MEblack. Of the top 20 hub genes in MEdarkgreen, 4 hub genes were involved in carbohydrate transport and metabolism, namely, BraA07g041160.3C, glucose-6-phosphate 1-dehydrogenase 3 (At1g24280), bifunctional enolase 2/transcriptional activator (*ENO2*), and 2,3-bisphosphoglycerate-independent phosphoglycerate mutase 1 (*PGM1*). In the MEgreenyellow of ‘JWW’, genes related to carbohydrate transport and metabolism pathways were also enriched, and the expression levels were notably higher than that of the control treatment, indicating that carbohydrate transport and metabolism may play an important role in the vernalization of Chinese cabbage. In MEpurple, 2 of the 20 hub genes encoded proteins and were enriched in the starch and sucrose metabolism pathway: trehalose-phosphate phosphatase A (*TPPA*) and sucrose synthase 1 (*SUS1*). Importantly, *SUS1* participates in flower development. Three WRKY family genes existed as hub genes in MEblack, including WRKY transcription factor 18 (*WRKY18*), transcription factor 25 (*WRKY25*), and transcription factor 57 (*WRKY57*), of which, *WRKY25* participated in the plant-pathogen interaction pathway. Among the top 20 hub genes in MEblack, 3 important genes were related to plant flowering, of which phophorbide a oxygenase (*PAO*) participated in flower development, protein twin sister of FT (*TSF*) regulated flower development and participated in photoperiodism and flowering, and NAC transcription factor 29 (*NAC029*) regulated flower development. The expression levels of these 3 genes across the ‘XBJ’ vernalization periods were significantly higher than those in the control treatment.

### Validation of representative flower development-related hub genes expression

**Fig. 10:**
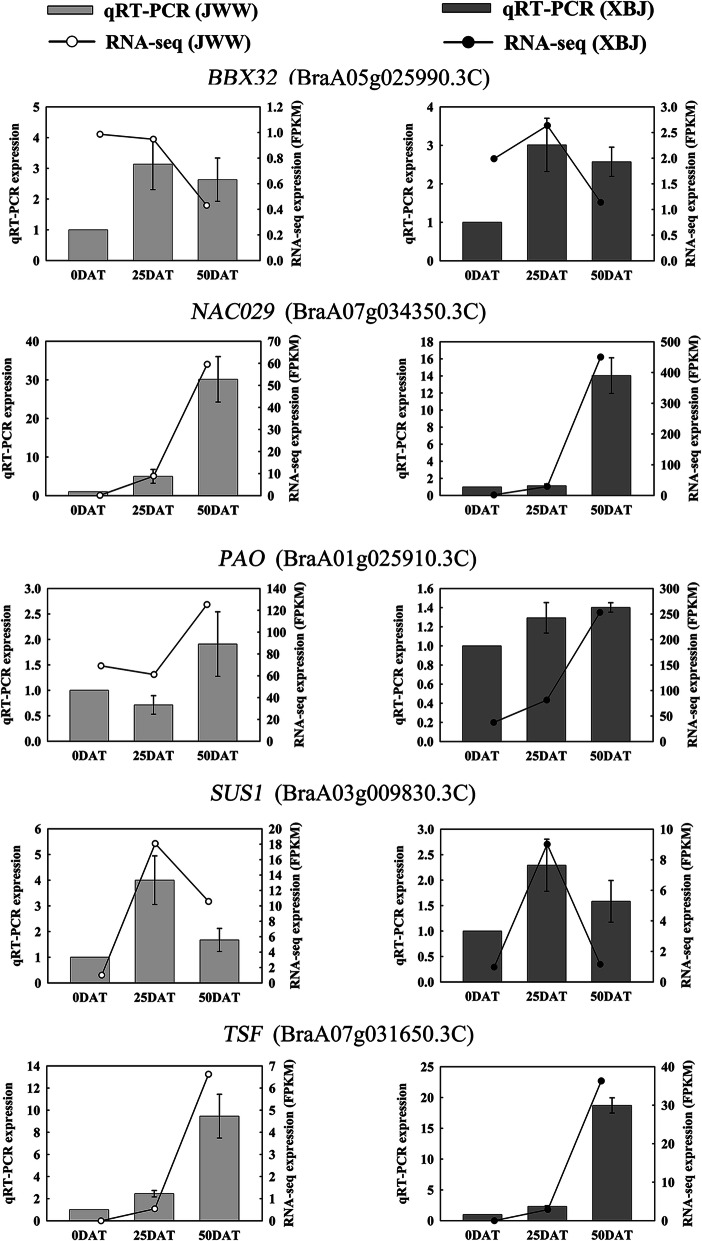
qRT-PCR verification of the hub genes related to flower development. The bar and line graphs represent the qRT-PCR and RNA-Seq data, respectively. Data are presented as mean ± standard error (SE)

Five genes, *BBX32, SUS1*, *PAO*, *TSF*, and *NAC029*, correlated with flower development and were selected for verification by qRT-PCR. The RNA-Seq and qRT-PCR results were consistent (Fig. 10), indicating the reliability of high-throughput transcriptome sequencing. Compared with 0 DAT, the expression of *SUS1* and *NAC029* in ‘JWW’ at 25 DAT and 50 DAT were higher than in ‘XBJ’. The expression of *TSF* and *BBX32* in ‘XBJ’ at 25 DAT and 50 DAT were higher than in ‘JWW’.

## Discussion

### Formation of specific co-expression networks using two bolting Chinese cabbage accessions and the WGCNA method

Phenotypic and molecular event-based RNA-Seq transcriptome analysis and WGCNA are powerful research methods [21–23]. WGCNA is a progressive analysis method in which variable genes are divided into co-expression modules through an unsigned network based on the gene expression patterns identified by RNA-Seq. Each module is then correlated with various traits and the gene “hubness” of each module builds the relationship between the positions of a single gene [24]. The eigengenes and hub genes of each module facilitate the establishment of the relationship between co-expressed gene clusters and concentrated traits in order to obtain clear expression patterns and screen candidate genes.

In this experiment, 2 Chinese cabbage accessions contained 21 RNA-Seq sample data points, respectively. Given that the expression of a large group of genes was affected by vernalization, WGCNA was used to construct a gene co-expression network to identify differences between modules (Fig. 2). The goal was to uncover the response mechanism of Chinese cabbage across different vernalization time periods and identify key genes. To our knowledge, there are no reports on the gene interaction networks of Chinese cabbage vernalization across different time periods. Therefore, based on the WGCNA gene co-expression network in this study, the responses of the 2 Chinese cabbage accessions to different vernalization stages were systematically analyzed at the transcriptome level.

### Enrichment of different modules based on the transcriptomic differences of two Chinese cabbage accessions

Several modules that were highly related were selected for further analysis and discussion (Figs. 3 and 4). Based on the functions predicted by the modules of genes with known biological functions, the characteristics of the 2 Chinese cabbage accessions under vernalization and control treatments were analyzed and determined in order to find the reasons for their different bolting processes.

Photosynthesis, chlorophyll biosynthetic, cytokinin, and karrikin-responsive biological functional terms were enriched in the two most highly correlated modules of the 2 Chinese cabbage accessions at 0 DAT: MEbrown and MEturquoise. The contribution of photosynthesis to vegetative growth depended, to a large extent, on leaf area, chlorophyll content per leaf area, and chloroplast lifespan [25, 26]. Cytokinins are a class of hormones that regulate both the division cycle and meristem homeostasis [27, 28]. Karrikin is a plant growth regulator that promotes germination and seedling photomorphogenesis [29]. At 0 DAT, the 2 Chinese cabbage accessions were in the vegetative growth stage, thus, they exhibited obvious and consistent performance in terms of photosynthesis, chlorophyll biosynthetic, cytokinin, and karrikin. Using the vernalization of two Chinese cabbage accessions at 0 DAT as a starting period, the different performances of the two could be better analyzed and the reasons for their different bolting performances explored.

In flowering plants, includingg the model plant *Arabidopsis thaliana*, the shoot apical meristem (SAM) is the key determinant of overall morphogenesis [30]. We found that among highly correlated modules at 25 DAT, the MEpurple of ‘XBJ’ was more enriched in shoot system morphogenesis functional term than MEgreenyellow of ‘JWW’. Futhermore, at 25 DAT vernalization caused ‘XBJ’ to rapidly bolt and flower. This indicated that 25 DAT was a well-chosen treatment period for ‘XBJ’. Another finding was that MEpurple was enriched in many polysaccharide and sugar metabolism terms. The formation of SAM is controlled by the growth of plant cells, and the growth of plant cells is mainly controlled by the cell wall. The cell wall is a rigid structure composed primarily of polysaccharides that surround the cells and connect them together in a biological continuum [31]. This result may be explained by the formation of SAM in ‘XBJ’, which requires polysaccharides to regulate the elasticity of the cell wall to ensure cell elongation and growth. ‘XBJ’ generated energy under vernalization to ensure its successful transition from vegetative to reproductive growth. Judging from the 25 DAT enriched terms, compared with late-bolting ‘JWW’, early-bolting ‘XBJ’ was more susceptible to changes in shoot system morphogenesis and many polysaccharide and sugar metabolism functional terms under vernalization, which then promoted faster bolting and flowering.

MEdarkgrey, which was highly related to rapid flowering at 35 DAT in ‘JWW’, was enriched in the biological functional terms response to chitin, ABA-activated signaling pathway, and ethylene-activated signaling pathway. A previous study demonstrated that treatment with chitosan, a chitin derivative, in potted freesia plants caused early flowering and more flowers [32]. Another study demonstrated that endogenous ABA promoted bolting and flowering in plants after the promotion of *FT* and other related genes [33]. The ABA-activated signaling pathway could have been enriched during the vernalization of ‘JWW’, indicating that endogenous ABA played a certain role in the promotion of flowering in ‘JWW’. The effect of ethylene on floral transition is a complex biological process, as ethylene regulates this process by cooperating with other hormones or signal transduction pathways [34]. This finding corresponds with the ethylene-activated signaling pathway, which was enriched at 35 DAT in ‘JWW’. These biological functional terms were enriched in MEdarkgrey in ‘JWW’ and may be the key determinants of late-bolting Chinese cabbage floral transition.

In previous studies, age-dependent leaf senescence was found to be affected by developmental processes such as flowering, and the age-dependent leaf senescence phenotypes of circadian clock mutants showed significant correlation with flowering time [35, 36]. We found an interesting phenomenon in that the MEblack module, which was highly correlated with ‘XBJ’ at 50 DAT, was enriched with terms related to senescence, especially the positive regulation of leaf senescence, while in ‘JWW’ MEblue at 35 DAT at 25 °C, the negative regulation of leaf senescence term was enriched. Fifty DAT was the longest time for vernalization. At this time, early-bolting ‘XBJ’ had transitioned from vegetative growth to reproductive growth. While 35 DAT at 25 °C ‘JWW’ exhibited vegetative growth under normal growth conditions, leaves grew normally and the senescence phenomenon was in a state of resistance. With this finding, we speculated that leaf senescence was also a signal that promoted bolting and flowering of Chinese cabbage. This also laid the groundwork for our future research on the effect of vernalization on leaf senescence and flowering.

In summary, we tried to find the biological function of two different bolting Chinese cabbage accessions during the vernalization process: the early-bolting ‘XBJ’ could bolt and flower faster at 25 DAT, which was promoted by the shoot system morphogenesis and polysaccharide and sugar metabolism, while late-bolting ‘JWW’ enriched chitin, ABA, and ethylene-activated signaling pathways at 35 DAT, indicating that these regulatory pathways may promote bolting resistance in Chinese cabbage. An interesting finding was that the regulation of leaf senescence was found in the 2 Chinese cabbage-related modules, indicating that leaf senescence may be related to bolting and flowering.

### Analysis of hub genes enriched in two Chinese cabbage accessions during vernalization

WGCNA was used to construct the gene co-expression networks of 2 Chinese cabbage accessions and analyze the modules that were highly related to their vernalization periods. The top 20 hub genes with the highest correlation relationships among these modules were identified to further analyze key candidate vernalization genes for Chinese cabbage with different bolting performances.

Amino acids are important constituents of proteins that play important roles in many pathways of the plant body, acting as biological stimulants under abiotic and biotic stress [37, 38]. Carbohydrates play a vital role in plant growth, reproduction, and flowering [39]. In this study, three hub genes, *MTHFR2*, *CPRD49*, and *AAP8*, were enriched in amino acid transport and metabolism pathways. Three other hub genes, *endoglucanase 10*, *BXL4*, and *BXL5*, were enriched in carbohydrate transport and metabolism pathways in ‘JWW’ MEgreenyellow. MTHFR is the least well-known enzyme in the folate-mediated 1-carbon metabolism of plants. MTHFR reactions in plants metabolize the methyl group 5,10-methylenetetrahydrofolate into serine, sugar, and starch [40]. *AAP8* has been studied in seeds and siliques as an amino acid transporter and was specifically expressed in mature siliques [41]. Previous studies demonstrated that beta-D-xylosidase was widely expressed in plant flowers, siliques, and the SAM [42, 43]. The high expression levels of *MTHFR2*, *AAP8*, *BXL4*, and *BXL5* during the vernalization of ‘JWW’ indicated that they were affected by vernalization and may have had an auxiliary promotion effect on the floral transformation of ‘JWW’. Three glycosyl transferase family genes, including two *GATL10* genes and one *GATL17* gene, were the main hub genes of ‘JWW’ MEdarkgrey. This finding was consistent with the results obtained from the GO biological function analysis of MEdarkgrey, indicating that sugar metabolism at 35 DAT in ‘JWW’ played an important role and promoted flower transformation. WRKY proteins, an important transcription factor superfamily involved in plant development and stress responses, have been studied in monocotyledonous and dicotyledonous plants [44]. In this study, three WRKY genes, *WRKY18*, *WRKY25*, and *WRKY57*, were identified in the hub genes of ‘XBJ’ MEblack. WRKY genes promote defense-related gene expression and disease resistance [45–47]. Vernalization is a form of low-temperature stress for Chinese cabbage. In this study, under the vernalization treatment, WRKY-related genes were enriched in ‘XBJ’ at 50 DAT.

Additionally, five genes related to flower development, *BBX32*, *SUS1*, *PAO*, *TSF*, and *NAC029*, were expressed in ‘JWW’ MEdarkgrey and ‘XBJ’ MEpurple and MEblack. B-box (BBX) zinc finger proteins play critical roles in both vegetative and reproductive plant development [48]. A previous study proved that *BBX32* in *Arabidopsis* is a clock gene that interacts with *COL3* and enables *COL3* to bind to the *FT* promoter, thereby promoting the transcriptional regulation of flowering time [49]. A separate study demonstrated that *BrBBX32* binds to *BrAGL24* in Chinese cabbage through the B-box domain, which regulates flowering time [50]. In this study, *BBX32* was enriched in ‘JWW’ at 35 DAT, indicating that vernalization induced *BBX32* expression and promoted the flowering transition of Chinese cabbage. A previous study showed that sucrose levels increased in the leaves and SAM of *Arabidopsis* exposed to strong radiation, thereby promoting bolting and flowering by increasing *FT* expression and inducing *SUS1* expression [51]. Sugar levels regulate plant flowering [52, 53]. In this study, vernalization induced *SUS1* levels, indicating that *SUS1* can be used as a candidate gene for Chinese cabbage vernalization. *TSF* is the closest homologue of *FT* and transgenic plants that overexpress *TSF* exhibit premature flowering [54]. The high expression levels of *TSF* in MEblack, which was highly correlated with ‘XBJ’ at 50 DAT, indicated that ‘XBJ’ began reproductive growth at this time; *TSF* was also continuously expressed. *PAO* is a chloroplast envelope-bound Rieske-type iron-sulfur oxygenase. The degradation of chlorophyll in *Arabidopsis* is related to *PAO* activities [55, 56]. In this study, the expression of *PAO* reached its maximum level at 50 DAT in both Chinese cabbage accessions, indicating that Chinese cabbage was gradually aged during vernalization. NAC family transcription factors play a role in leaf senescence [57]. One previous study found that multiple *NACs* played regulatory roles in flowering [58]. In this study, under the vernalization treatment, the expression of *NAC029* in both Chinese cabbage accessions increased and was significantly upregulated in ‘XBJ’. This finding demonstrated that the early-bolting ‘XBJ’ accession could more quickly adapt to vernalization and after the formation of the SAM, its leaves gradually aged. When we performed biological functions on the highly correlated modules of two Chinese cabbage treatment periods, we found that the positive and negative regulation of leaf senescence was enriched in the vernalization and non-vernalization periods, which was consistent with the high expression of *PAO* and *NAC029* found here. This collectively proved that the vernalization process and the aging mechanism have a connection, although whether it promotes the flowering transition remains to be determined.

## Conclusion

Vernalization as an important trait that is directly linked to production potential. It is necessary to elucidate the regulatory mechanisms involved in differently-bolting Chinese cabbage varieties. In this study, a WGCNA was conducted using RNA data from two Chinese cabbage accessions that bolt differently in order to reveal the key pathways and hub genes that cause bolting and flowering during vernalization. The results revealed that shoot system morphogenesis and polysaccharide and sugar metabolism induce early-bolting ‘XBJ’ to bolt and flower faster; chitin, ABA and ethylene-activated signaling pathways were enriched in late-bolting ‘JWW’; and leaf senescence and carbohydrate metabolism pathways were found to be enriched in the two Chinese cabbage-related modules, indicating that these may be related to bolting and flowering. Additionally, five genes related to flower development, *BBX32* (binds to the *FT* promoter), SUS1 (increases *FT* expression), *TSF* (the closest homologue of *FT*), *PAO*, and *NAC029* (plays a role in leaf senescence), revealed different vernalization mechanisms. The findings of this study provide a comprehensive overview of vernalization-related gene networks in Chinese cabbage and uncovered candidate hub genes in the vernalization process that can be utilized in future breeding research.

## Methods

### Chinese cabbage accessions

**Fig. 11:**
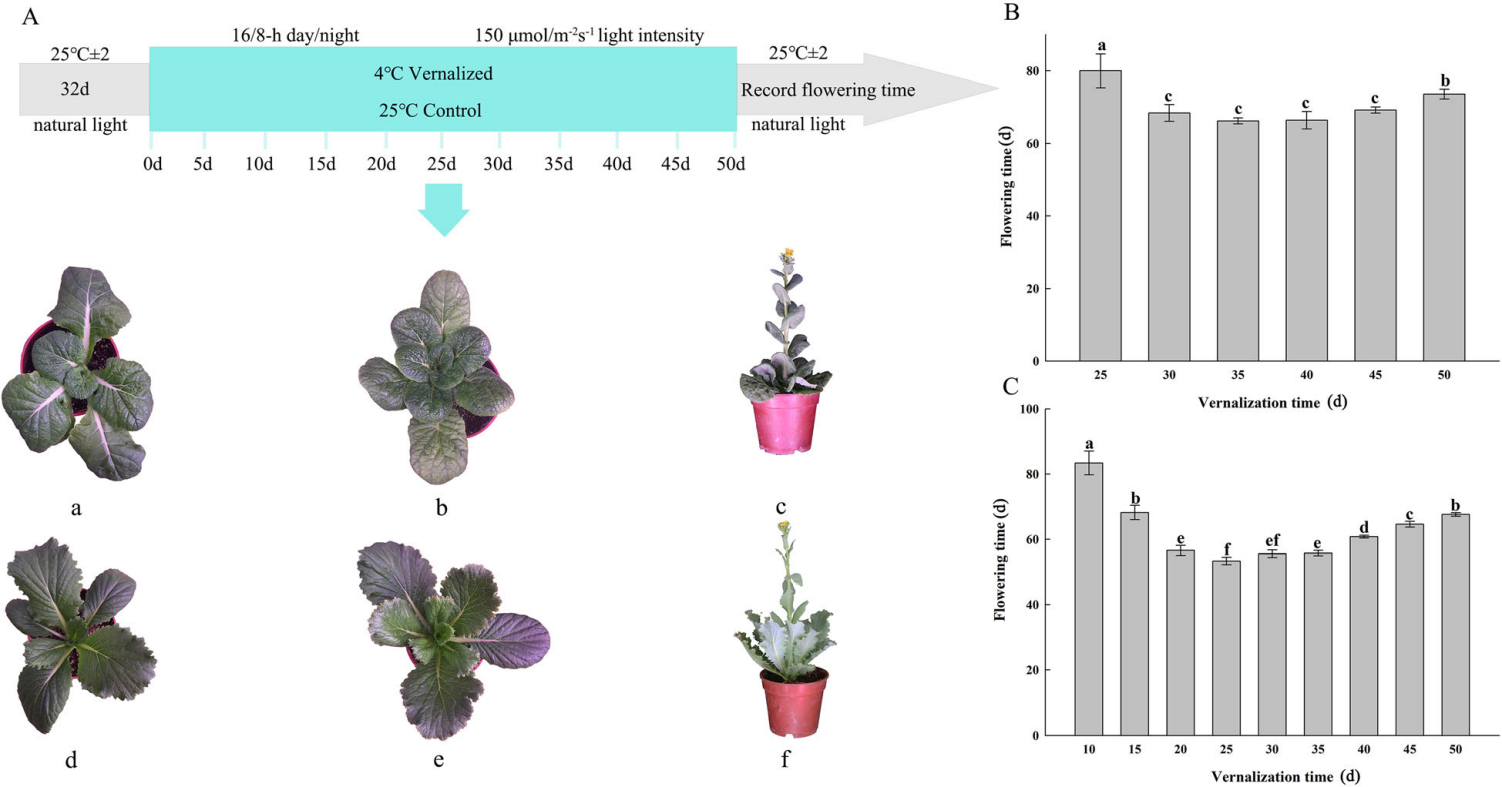
**a** Treatment process of two Chinese cabbage accessions: (**a**), (**b**), and (**c**) are ‘JWW’; (**d**), (**c**), and (**f**) are ‘XBJ’; (**a**) and (**d**) are before vernalization (0 DAT); (**b**) and (**c**) are during vernalization (25 DAT); (**c**) and (**f**) are after flowering. **b** ‘JWW’ flowering time promoted by vernalization. **c** ‘XBJ’ flowering time promoted by vernalization

Two Chinese cabbage accessions, the late-bolting Jin Wawa (JWW) and early-bolting Xiao Baojian (XBJ), were provided by the Chinese Academy of Agricultural Sciences located in Beijing, China. The two materials were highly inbred lines. After plants were grown in a nursery greenhouse under normal conditions for 32 d, the vernalization experiment began and lasted for 50 d (Fig. 11a). The treatment conditions were 4 °C for the vernalization treatment and 25 °C for the control treatment.

### Sample selection and RNA sequencing

Based on the timing of flowering caused by vernalization (Fig. 11b; c), the following samples were collected for RNA-Seq: from ‘JWW’, selected samples included J1 (0 days after treatment (DAT)), J2 (25 DAT), J3 (30 DAT), J4 (35 DAT), J5 (45 DAT), J6 (50 DAT), and JCK (35 DAT 25 °C), and from ‘XBJ’, selected samples included X1 (0 DAT), X2 (10 DAT), X3 (15 DAT), X4 (25 DAT), X5 (40 DAT), X6 (50 DAT), and XCK (25 DAT 25 °C). Three biological replicates were collected for each sample. The vernalization treatment, sample collection method, RNA-seq period selection, and the detailed methods used for data processing are described in our previous study [59].

### Gene co-expression network construction and visualization

The RNA-Seq data were analyzed to construct gene co-expression networks using the R package, WGCNA [60]. Based on the following criteria, Fragments Per Kilobase of transcript per Million mapped reads (FPKM) ≥ 1, and a variation of FPKM: cv ≥ 0.5 and cv ≤ sd (genes number)/mean (genes number) (‘sd’ represents the standard deviation of the sample, and ‘mean’ represents the calculated average of the sample),genes of the 2 Chinese cabbage accessions were screened for co-expression network construction. From ‘JWW’, 5748 co-constructed genes were screened out. The following parameters were used to identify each gene module: weighted network, unsigned; hierarchical clustering tree, dynamic hybrid tree cut algorithm; power, 5; and minimum module size, 30; minimum height for merging modules, 0.29995. From ‘XBJ’, 5527 co-constructed genes were screened out. The following parameters were used to identify each gene module: weighted network, unsigned; hierarchical clustering tree, dynamic hybrid tree cut algorithm; power, 5; minimum module size, 30; and minimum height for merging modules, 0.3131.

To describe the most common gene expression models in each module, module eigengenes were used. Module eigengenes are the first major components of the expression matrix and are used to summarize the module overview and feature data. Pearson’s correlation coefficients were used to calculate the correlation between the module characteristic genes and the degree of vernalization of the two Chinese cabbage accessions. A heat map was drawn according to the correlation coefficients. The depth of color represents the correlation between the module and the degree of vernalization.

### Analysis of hub genes in the gene co-expression network

Hub genes are good representatives of each co-expression module and have important biological significance in the system analysis. Hub genes are genes with the most connection points in each module, and their height is represented by the kME value. The kME value is based on the Pearson correlation coefficient between the expression level and module eigengenes. The kME and eigengene connectivity of each gene are calculated by signedKME, which includes the edge and node characteristics. The genetic network map, which was drawn according to the kME values, was created using Cytoscape software [61].

A gene ontology (GO) enrichment analysis was conducted on the genes using GOseq R software [62] and ClueGO [63]. The *Brassica* Database v3.0 IDs [64] were used as search queries for the GOseq R software annotations; GO terms with FDR values < 0.01 were selected for output. The TAIR10 IDs were used as search queries for the ClueGO annotations. ClueGO is a cytoscape plugin for visualizing large gene clusters in a functionally grouped network that can analyze both single clusters and compare them based on their specificity and the same aspects of multiple cluster functions. The ClueGO network was set to ‘medium’ and its connectivity was based on a kappa score of 0.4. GO terms with *p* ≤ 0.01 were considered to be significant. Other parameters were based on the original ClueGO values. Gene functions were annotated based on the Swiss-Prot, KOG/COG, KO, Pfam, and Nr NCBI databases.

### Quantitative Real-Time PCR (qRT-PCR) and the evaluation of candidate hub gene expression

Five hub genes were selected to evaluate their expression levels by qRT-PCR analysis. Gene-specific primers were designed using Primer v5.0. *Actin* was used as an internal control for gene expression (Table S7). The Bio-Rad CFX96 RT-PCR Detection system (Bio-Rad, Hercules, CA, USA) and SYBR Green II PCR Master mix (Takara, Nojihigashi, Kusatsu, Japan) were used for the qRT-PCR reactions. The gene expression data were analyzed using the 2^-ΔΔCt^ method [65]. SPSS v19.0 (SPSS, Chicago, IL, USA) was used to conduct a one-way analysis of variance (ANOVA) with Duncan’s multiple range post-hoc test and a significance threshold of *p* < 0.05. Results were visualized using Sigmaplot v10.0 (Systat Software Inc., San Jose, CA, USA).

## Supplementary Information

**Additional file 1: Table S1.** Log_2_FPKM values of the 5748 variable genes in ‘JWW’ (J for ‘JWW’). **Table S2.** Log_2_ FPKM values of the 5527 variable genes in ‘XBJ’ (X for ‘XBJ’). **Table S3.** Significant GO terms for each gene set of ‘JWW’. **Table S4.** Significant GO terms for each gene set of ‘XBJ’. **Table S5.** Annotation information of the hub genes of each module in ‘JWW’. **Table S6.** Annotation information of the hub genes of each module in ‘XBJ’. **Table S7.** qRT-PCR specific primers for hub gene related to flower development.

## Data Availability

The raw data have been submitted under BioProject number PRJNA615255 to the Sequence Read Archive (SRA) database at NCBI (https://www.ncbi.nlm.nih.gov/sra/PRJNA615255).

## Abbreviations

JWW: Jin Wawa
XBJ: Xiao Baojian
DAT: Days after treatment
RNA-seq: RNA sequencing
FPKM: Fragments Per Kilobase of transcript per Million mapped reads
WGCNA: Weighted gene co-expression network analysis
GO: Gene ontology
ABA: Abscisic acid
DEGs: Differentially expressed genes
PCA: Principal components analysis
PC1: First principal component
PC2: Second principal component
MTRs: Module-trait relationships
SAM: Shoot apical meristem
qRT-PCR: Quantitative real-time PCR
